# Genome Survey Sequencing of *Luffa Cylindrica* L. and Microsatellite High Resolution Melting (SSR-HRM) Analysis for Genetic Relationship of *Luffa* Genotypes

**DOI:** 10.3390/ijms18091942

**Published:** 2017-09-11

**Authors:** Jianyu An, Mengqi Yin, Qin Zhang, Dongting Gong, Xiaowen Jia, Yajing Guan, Jin Hu

**Affiliations:** Seed Science Center, Institute of Crop Science, College of Agriculture and Biotechnology, Zhejiang University, 866 Yuhangtang Road, Hangzhou 310058, China; anjianyu@live.cn (J.A.); yinmengi@icloud.com (M.Y.); 21616042@zju.edu.cn (Q.Z.); 21616033@zju.edu.cn (D.G.); 21516035@zju.edu.cn (X.J.); jhu@zju.edu.cn (J.H.)

**Keywords:** genome survey, genomic SSR markers, SSR-HRM, genetic diversity, *Luffa* (*L. cylindrica*)

## Abstract

*Luffa cylindrica* (L.) Roem. is an economically important vegetable crop in China. However, the genomic information on this species is currently unknown. In this study, for the first time, a genome survey of *L. cylindrica* was carried out using next-generation sequencing (NGS) technology. In total, 43.40 Gb sequence data of *L. cylindrica*, about 54.94× coverage of the estimated genome size of 789.97 Mb, were obtained from HiSeq 2500 sequencing, in which the guanine plus cytosine (GC) content was calculated to be 37.90%. The heterozygosity of genome sequences was only 0.24%. In total, 1,913,731 contigs (>200 bp) with 525 bp N_50_ length and 1,410,117 scaffolds (>200 bp) with 885.01 Mb total length were obtained. From the initial assembled *L. cylindrica* genome, 431,234 microsatellites (SSRs) (≥5 repeats) were identified. The motif types of SSR repeats included 62.88% di-nucleotide, 31.03% tri-nucleotide, 4.59% tetra-nucleotide, 0.96% penta-nucleotide and 0.54% hexa-nucleotide. Eighty genomic SSR markers were developed, and 51/80 primers could be used in both “Zheda 23” and “Zheda 83”. Nineteen SSRs were used to investigate the genetic diversity among 32 accessions through SSR-HRM analysis. The unweighted pair group method analysis (UPGMA) dendrogram tree was built by calculating the SSR-HRM raw data. SSR-HRM could be effectively used for genotype relationship analysis of *Luffa* species.

## 1. Introduction

*Luffa*, or sponge gourd, belonging to the Cucurbitaceae family, is a diploid species with 26 chromosomes (2*n* = 26) and a cross pollinated crop [1]. *Luffa cylindrica* (L.) Roem. is one of the most important cultivar species and is mainly planted in tropical and subtropical areas, such as China, Thailand, India, Malaysia, etc. If fruits are harvested at young stage of development, they can be eaten as an edible vegetable, which contains abundant bioactive substances such as alkaloids, flavonoids, sterols, glycosides and glycoprotein to benefit human health [2,3]. When *Luffa* is fully ripened, the tough fibrous netting from the matured fruit can be used in the bath and kitchen, or as marine steam engine filters and industrial raw materials [4]. *Luffa acutangula* (L.) Roem. is another species of *Luffa* genus, which is closely related with *Luffa cylindrica* (L.) Roem. [1].

Although many studies on *Luffa* germplasm resources and conventional breeding have been performed, genetic studies are still in their infancy. Currently, no genome survey sequences on *Luffa* have been reported (as at July 2017), Moreover, only 372 DNA and RNA sequences (https://www.ncbi.nlm.nih.gov/nuccore/), 41 sequence sets from phylogenetic and population study (https://www.ncbi.nlm.nih.gov/popset/) and one expressed sequence tag (EST) (https://www.ncbi.nlm.nih.gov/nucest/) could be found in National Center for Biotechnology Information (NCBI). The narrow genetic and genomic resources obviously limited the breeding improvement of *Luffa*.

The recent development of next-generation sequencing (NGS) technologies has produced a large amount of available sequence data. Genome survey sequencing via NGS is an important and cost-effective strategy in generating extensive genetic and genomic information relating to the metabolism and development of organisms [5]. Therefore, to investigate and provide a genomic resource of *Luffa* for further study, genome survey of *L. cylindrica* was conducted using NGS technology. These results would be useful for crop improvement programs and better utilization of genomic information in the future [6].

In addition, because of the advantages including decent reproducibility, co-dominance, relative abundance and simplicity, SSR markers have become one of the most useful tools for genetic diversity and linkage mapping analysis. Genomic SSRs and EST SSRs are considered complementary for plant genome mapping [7]. Recently, 1046 pairs of EST-SSR markers were synthesized and verified through transcriptome sequencing in sponge gourd [1,8]. EST-SSRs are useful for genetic analysis; however, their primary limitations are relatively low polymorphism and high possibility of no gene-rich regions in the genome. In contrast, genomic SSRs are highly polymorphic and tend to be widely distributed throughout the genome, resulting in better map coverage [9]. Thus far, no genomic sequence-based markers are available for *Luffa*.

Meanwhile, high resolution melting (HRM), a sensitive mutation detecting method, has been identified as a powerful, efficient and cost-effective method to analyze genetic variation [10]. It was considered as an evolution of real-time polymerase chain reaction (PCR) technology. During the process of PCR, fluorescent dyes would insert into double-stranded DNA, and accumulate until the end of PCR. Then, with the temperature increasing 0.1 °C or more per one second from annealing temperature (~60 °C) to relatively high temperature (~95 °C), fluorescence dye separated from DNA, and fluorescence signal was detected at the same time to analyze the *Tm* values, melting curves and other information. Discrimination of the target amplicon from non-specific products can be done by measuring the difference between *Tm* values, and the shape of HRM curves [11]. Each HRM curve of amplicon had its own accurate characteristic, which depends on GC content, amplicon length and sequence of the nucleotide sequence [12]. The differences between different amplicons need to be determined after further normalization [13]. Based on the HRM curves, amplicons can be identified even with the same *Tm* values, and be classified to several categories according to their normalized melting curves and difference plots. Moreover, it allows detection of sequence variants without sequencing or hybridization procedures [14]. The combined analysis HRM and SSR has been used to differentiate highly similar cultivars of sweet cherry [11], lentils (*Lens culinaris*) [15] and *Olea europaea* [16]. However, the relevant study on *Luffa* has not yet been reported.

In this study, *L. cylindrica* genome sequence produced by genome survey sequencing was reported, which was used to develop a set of new genomic SSR markers of *Luffa*. The HRM technique efficiency for identifying SSRs in PCR amplifications was assessed and the SSR-HRM method was used to discriminate different *Luffa* species and cultivars.

## 2. Results

### 2.1. Genome Sequencing and Sequence Assembly

Based on the genome sequence data, 43.40 Gb clean reads were generated from the small-insert (220 bp) library. The approximate 54.94× coverage (Table 1) was much better than 30X coverage, which indicated successful assembly [17]. Assembly with K-mer 75 by the SOAPdenovo produced scaffolds with the N_50_ of ~807 bp, and a total length of ~885.01 Mb (Table 2).

### 2.2. Genome Size Estimation, GC Content and Genome Survey

**Figure 1:**
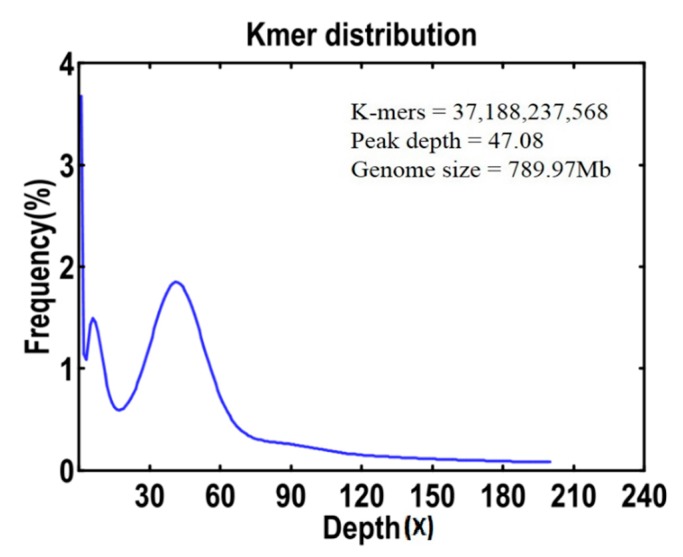
The distribution of K-mer (*K* = 19) analysis based on the whole genome shotgun data in *Luffa cylindrica* L. (The peak K-mer frequency was 47.08. Genome size was estimated with the formula: estimated genome size = K-mer count/Peak of the depth distribution).

**Figure 2:**
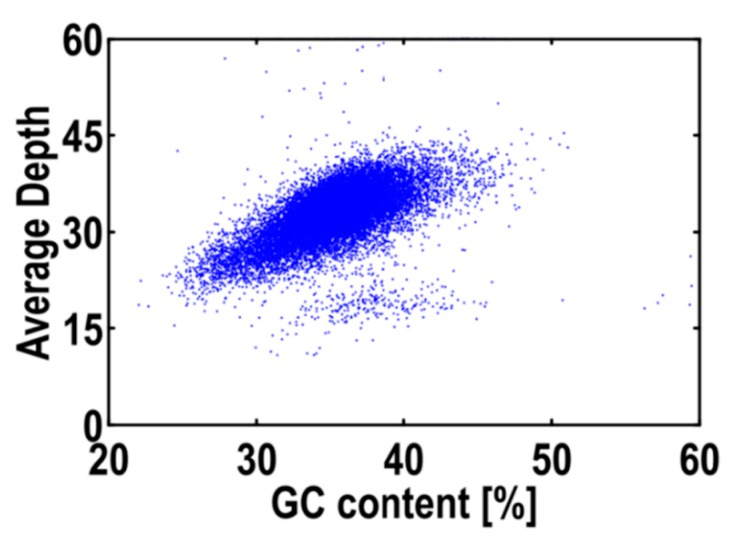
GC content and average sequencing depth of the *Luffa cylindrica* L. genome data used for assembly. (The *x*-axis was GC content percent across every 10-kb non-overlapping sliding window.)

For the 19-mer frequency distribution (Figure 1), the number of K-mers was 37,188,237,568, and the peak of depth distribution was 47.08. The estimated genome size was 789.97 Mb. Similarly, the minor peak at the position of the integer multiples of the main peak indicated a certain repeat rate, and the position at half of the main peak indicated the heterozygosis rate, which were 71.37% and 0.24% in *L. cylindrica* genome, respectively. *L. cylindrica* had a mid-GC content of 37.90% (Figure 2).

**Figure 3:**
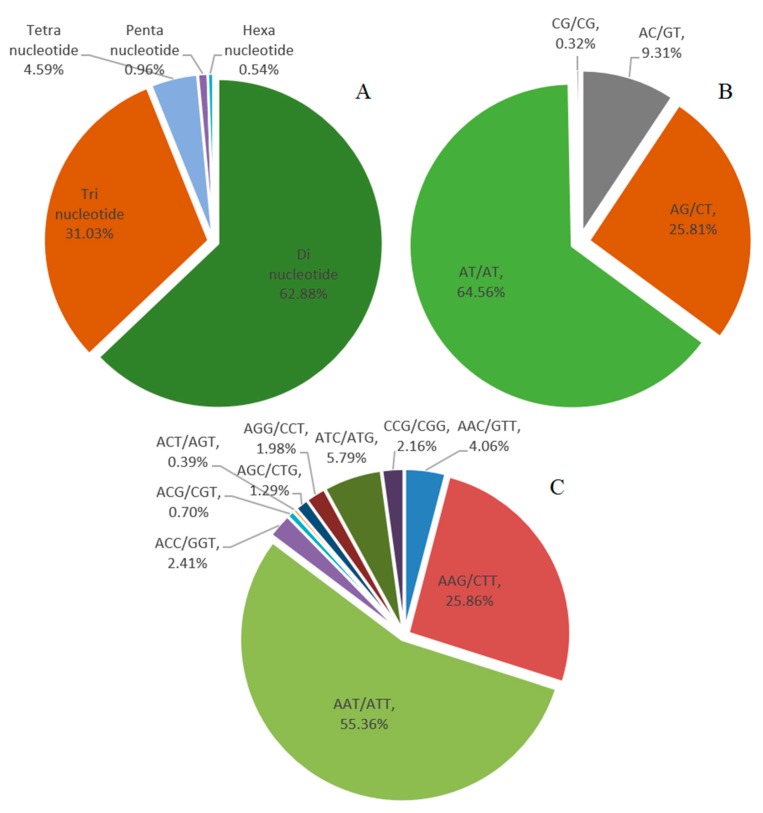
Identification and characteristics of simple repeat sequence (SSR) motifs: (**A**) frequency of different SSR motif repeat types; (**B**) frequency of different di-nucleotide SSR motifs; and (**C**) frequency of different tri-nucleotide SSR motifs.

From the 1,064,011,890 bp genome survey sequence, 431,234 SSRs were identified (Table 3). The motif length of SSR repeats (without mono-nucleotide) included 62.88% di-nucleotide, 31.03% tri-nucleotide, 4.59% tetra-nucleotide, 0.96% penta-nucleotide and 0.54% hexa-nucleotide repeats (Figure 3A). Within the di-nucleotide repeat motifs, the AT/AT accounted for 64.56%, AG/CT for 25.81%, AC/GT for 9.31% and CG/CG only for 0.32% (Figure 3B). The predominant tri-nucleotide motifs, AAT/ATT, AAG/CTT and ATC/ATG repeats, respectively, accounted for 55.36%, 25.86% and 5.79% (Figure 3C).

**Figure 4:**
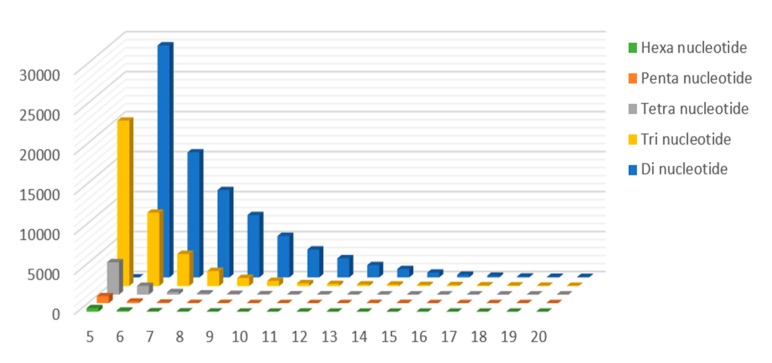
The distribution and frequency of SSR motif repeat numbers. (*x*-axis, SSR repeat numbers; *y*-axis, frequency of this SSR type; blue bars, di-nucleotide repeat motifs; yellow bars, tri-nucleotide repeat motif; gray bars, tetra-nucleotide repeat motifs; orange bars, penta-nucleotide repeat motifs; and green bars, hexa-nucleotide repeat motifs.)

SSR motifs categorized by their unit sizes and the number of repeats were summarized (Figure 4). The numbers of di-nucleotide and tri-nucleotide repeats were much more than the other four types. The frequency distribution eliminate range of SSR motif repeats among genomic SSR markers in *L. cylindrica* ranged 6–37 repeats for di-nucleotide, 5–25 for tri-nucleotide, 5–18 for tetra-nucleotide, 5–15 for penta-nucleotide, and 5–12 for hexa-nucleotide. The variations of repeat numbers decreased with increased motif length.

### 2.3. Genomic SSR Markers Development

**Figure 5:**
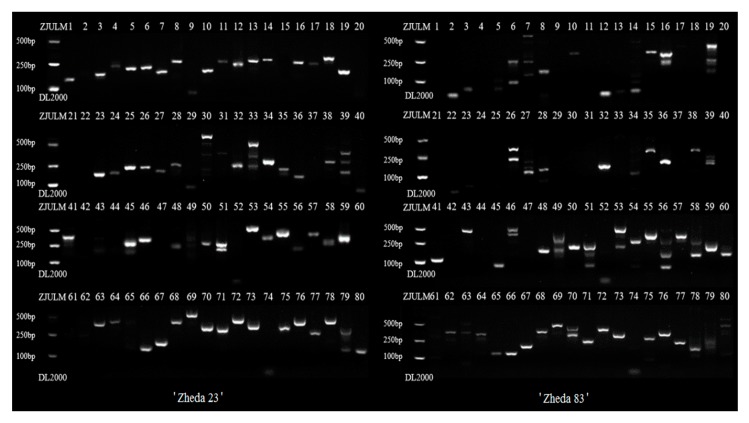
Result of PCR products on 3% agarose gel at 110V. (Two inbred cultivars, “Zheda 23” (*L. cylindrica*) and “Zheda 83” (*L. acutangula*), were tested by 80 *Luffa* genomic SSR markers).

Based on the genome survey of *L. cylindrica*, 80 genomic SSR primer pairs were designed and synthesized, which were mainly perfect five and six SSR motif repeats. These markers were tested using two inbred cultivars, “Zheda 23” (*L. cylindrica*) and “Zheda 83” (*L. acutangula*.). The hybrids between *L. cylindrica* and *L. acutangula* had strong heterosis effects [1]. The results in Figure 5 showed that 65 of 80 SSR loci were amplified in “Zheda 23”, and 59 of 80 in “Zheda 83”, in which, 51 common SSR loci were identified.

Nineteen of fifty-one identified SSR loci were chosen (Table 4) to perform HRM analysis, which were clear enough and could be amplified by both *Luffa* cultivars. Gene diversity ranged from 0.1913 to 0.8137, and the PIC at each locus ranged from 0.1730 to 0.7896 with an average of 0.5281.

### 2.4. Genetic Relationship Analysis by SSR-HRM

For genetic relationship analysis using SSR-HRM method, the genotype of each DNA sample was determined based on the shape of curves depicted by temperature-shifted melting curves and difference plots. In the analysis option of software, both delta*T*m discrimination and curve shape sensitivity were set up to 50%. Afterwards, the curves were analyzed, melting curves and difference plots were obtained, and these curves were clustered to several genotype groups. The corresponding accessions would be recorded with their genotype group number (Table 5).

**Figure 6:**
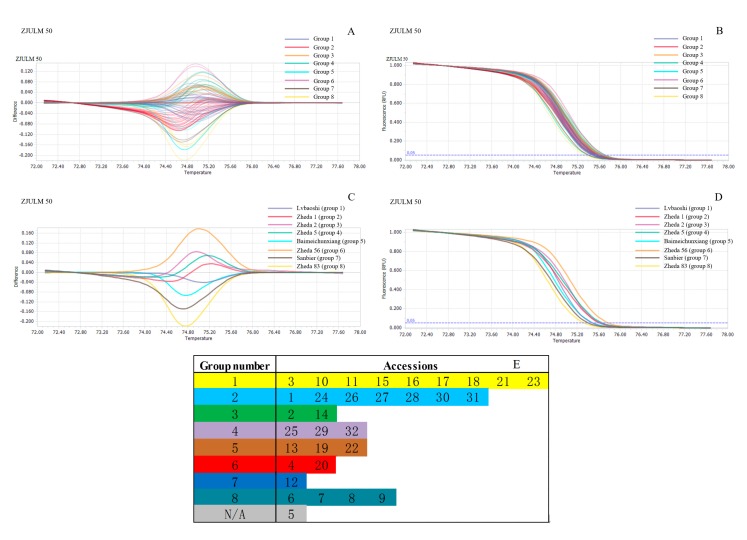
Difference plots and normalized HRM melting curve analyses obtained with the SSR marker ZJULM 50: (**A**) difference plots of 32 *Luffa* accessions (each amplicon with three repeats) by the genomic SSR marker ZJULM 50; (**B**) normalized HRM melting curve of 32 *Luffa* accessions (each amplicon with three repeats) by the genomic SSR marker ZJULM 50, where the same line color indicates the same genotype calculated by HRM analysis; (**C**) difference plots of the eight distinguished *Luffa* accessions by the genomic SSR marker ZJULM 50; (**D**) normalized HRM melting curve of the eight distinguished *Luffa* accessions by the genomic SSR marker ZJULM 50; and (**E**) genotype group number of each accession. Blue dotted line 0.05 in (**B**) and (**D**) meant Fluorescence (RFU) = 0.05.

For example, using the genomic SSR marker, Zhejiang University *Luffa* marker (ZJULM) 50, the difference plots of 32 accessions are shown in Figure 6A and normalized melting curves in Figure 6B. All these cultivars were obviously gathered into eight unique *Luffa* genotypes. The representative HRM genotype in each group is shown in Figure 6C,D, which could be easily distinguished visually by their difference plots and melting curves, such as “Zheda 2” (group 3) and “Sanbier” (group 7). Then, each accession was marked with its own genotype group number (Figure 6E) for further study. The 18 other SSR markers were performed similar to ZJULM 50, and finally the result of these 19 groups of SSR-HRM are presented in Table 5.

**Figure 7:**
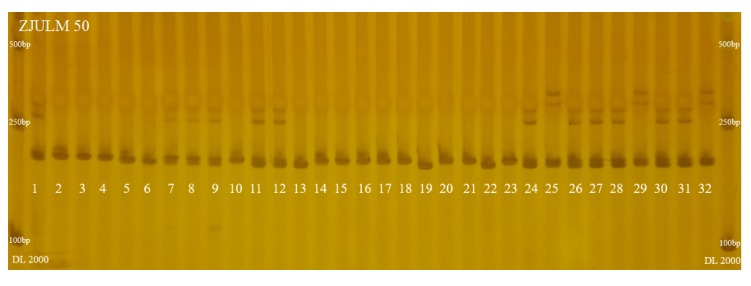
Results of PCR products obtained with the SSR marker ZJULM 50 on 6% denaturing polyacrylamide gel at 60 mA constant current (Nos. 1–32, 32 accessions, detailed explanation can be seen in Table 6; DNA Marker: DL2000).

**Figure 8:**
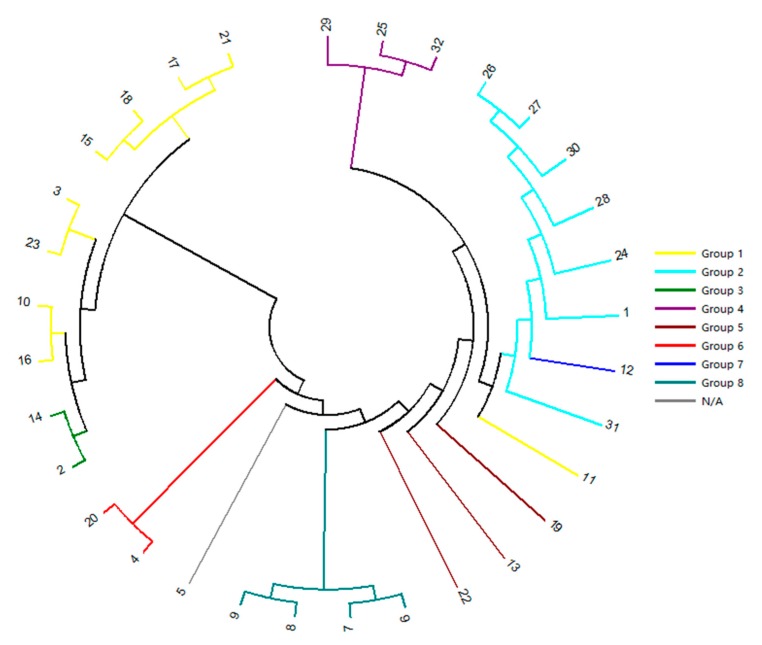
Evolutionary relationships of PCR products obtained with the SSR marker ZJULM 50 based on sequencing analysis. (Nos. 1–32, 32 accessions, detailed explanation can be seen in Table 7; the color of the branches is the same with the result of SSR-HRM group color in Figure 6E).

In addition, to evaluate the reliability and efficiency of SSR-HRM method, polyacrylamide gel electrophoresis (PAGE) and sequencing were carried out using the same PCR product obtained with ZJULM 50, thus the results can directly be compared with SSR-HRM results in Figure 6. The results of PAGE could only cluster those 32 accessions to four taxa (Figure 7). All PCR products obtained with ZJULM 50 were sequenced, and, to get the cluster result of these sequencing data, evolutionary relationships of those 32 accessions were measured by MEGA7, based on Neighbor-Joining method [18] (Figure 8).

**Figure 9:**
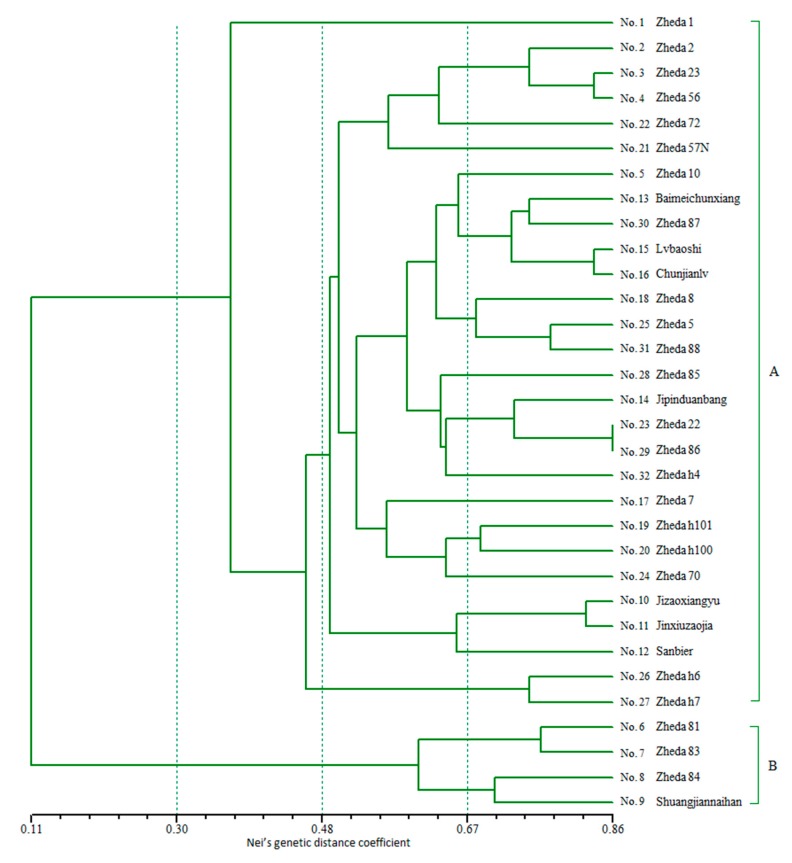
Dendrogram for 32 *Luffa* accessions derived from UPGMA cluster analysis obtained with the 19 genomic SSR markers using HRM analysis. Coefficient was based on Nei’s genetic distance coefficient [19].

According to these 19 group SSR-HRM genotype data (Table 5), dendrogram for 32 *Luffa* accessions were performed (Figure 9) based on Nei’s genetic distance coefficient [19], and then these 32 accessions were divided into two groups: Cluster A and Cluster B. Cluster A was comprised of 28 accessions belonging to *L. cylindrica*, and Cluster B included four accessions belonging to *L. acutangula*. The genetic distance coefficient between *L. cylindrica* and *L. acutangula* was 0.11, higher than 0.00 that was previously reported by Wu et al. [8]. According to cluster analysis above, the similarity among all these accessions ranged from 0.11 to 0.86.

## 3. Discussion

### 3.1. Characteristics of Luffa cylindrica L. Genome

The genomes of Cucurbitaceae family members *Cucumis melo* L. and *Cucumis sativus* L. have been reported. The genome size of *Cucumis melo* L. was 375 Mb, representing 83.3% of the estimated melon genome [20], while *Cucumis sativus* L. was only 243.5 Mb, 72.8% sequence anchored on chromosome [21]. From our genome survey data, the estimated genome size of *L. cylindrica* was 789.97 Mb using all of the clean data for K-mer analysis, which was almost two times of *Cucumis melo* L. and three times of *Cucumis sativus* L. Genome data of *L. cylindrica* can be improved by the additional sequencing of larger insert libraries to increase the contig and scaffold sizes. With the development of NGS technologies, genome sequencings of horticultural plants, such as *L. cylindrica*, not only help easily understand genome organization and critical gene associated with important traits, but also help conveniently design more highly polymorphic molecular markers for subsequent application in molecular breeding [22]. In addition, these data here contribute to genomic research of sponge.

The GC content affected the sequence bias directly [23]. More than 65% or less than 25% GC contents might cause sequence bias on the Illumina sequencing platform. Because genomic sequences obtained through high-throughput sequencing are not uniformly distributed across the genome. This systematic bias is a particular problem for techniques, thus seriously affecting genome assembly [24,25]. *L. cylindrica* had 37.90% GC content, which was higher than that of potatoes (34.8–36.0%) [26,27], while lower than that of *Rosa roxburghii* Tratt (~38.64%) [8], *Gracilariopsis lemaneiformis* (~48%) [9], human (~41%) and *Nasonia vitripennis* (~40.6%) [28].

From the 1,064,011,890 bp genome survey sequence, 125,094 SSRs without mono-nucleotide repeats were identified. Therefore, the distribution of SSRs in the genome of *L. cylindrica* was estimated to be about 117.57 SSRs per Mb, which is lower than the 135.5 SSRs per Mb in *Arabidopsis* [29]. Among the di-nucleotide repeat motifs, AT/AT accounted for 64.56% and was confirmed to be the most abundant type, followed by AG/CT, accounting for 25.81%. This was consistent with the results that AAT/ATT, AAAT/ATTT, AAAAT/ATTTT and AAAAAG/CTTTTTT were the most abundant repeat motifs in their SSR motif repeats, and they were all A/T rich motifs existing in *L. cylindrica.* This phenomenon was similar to other species such as *Brassica napus* [30], rice [31], peanut [32] and *Arabidopsis* [33], in which A/T rich motifs also performed a dominant role.

### 3.2. Genomic SSR Markers Development

In this study, 65/80 of genomic SSR markers could be amplified by “Zheda 23” (*L. cylindrica*) and 59/80 by “Zheda 83” (*L. acutangula*) based on agarose gel electrophoresis. Nineteen markers were used for SSR-HRM, and the other markers (Table 6), which could also be amplified by “Zheda 23” or “Zheda 83”, might be meaningful for further study such as QTL mapping. All of these genomic SSR markers were valuable for fingerprinting and genetic analysis of *Luffa*.

### 3.3. Genetic Relationship Analysis by SSR-HRM

Following the standard protocol of HRM, experiment data were obtained that could be analyzed by corresponding software. During analysis period, delta*T*m discrimination and curve shape sensitivity were two core parameters that significantly affected the clustering results. If the sensitivity were too high, the clustering results obtained with the same SSR marker would be diverse from each other, while, if the sensitivity were too low, it would lead to completely consistent results. These two situations would make clustering difficult to carry out. According to the experiment, 50% would be the appropriate level of these two parameters. In this case, the curves were analyzed, melting curves and difference plots were generated, and these curves were clustered to several genotype groups. The corresponding accessions was recorded with their genotype group number, and then these genotype results were analyzed with traditional SSR analysis software, such as PopGene, Power Marker, NTSYS, etc. It provided us an unusual experience of quickly identifying different accessions.

PAGE and sequencing are traditional methods to analyze PCR products. From the results of PCR products obtained with ZJULM 50 based on PAGE (Figure 7), these 32 accessions could be clustered into four taxa, while SSR-HRM (Figure 6) easily gathered those accessions into taxa with visual difference. As for the result of sequencing (Figure 8), more than nine taxa were demonstrated.

Comparing the result of SSR marker ZJULM 50 by PAGE, sequencing and SSR-HRM methods, many similarities and differences were found. For example, Nos. 25, 29 and 32 had a similar banding pattern in PAGE, and were also proven to be closely related in sequencing and SSR-HRM. A similar situation was found in Nos. 13, 19 and 22 accessions. In addition, Nos. 1, 7, 8, 9, 11, 12, 24, 26, 27, 28, 30 and 31 in PAGE have similar banding patterns, with one main belt and two incidental ones represented at the same level, thus these accessions were classified to one taxon. However, according to the sequencing result (Figure 8), Nos. 7–9 were clearly separated from the other nine accessions. This result was closer to the sample characteristics that Nos. 7–9 accessions were *L. acutangula*, but the other nine accessions belonged to *L. cylindrica*. The result of SSR-HRM (Figure 6E) was nearly the same with sequencing on this point, in which Nos. 1, 24, 26, 27, 28, 30 and 31 were identified to be same taxon, and Nos. 7–9 were classified to another taxon. In addition, as No. 6 belonged to *L. acutangula*, No. 6 showed a relatively close genetic relationship with Nos. 7–9 in sequencing and SSR-HRM. However, different banding patterns obviously misled the judgment of No. 6 in PAGE. Thus, it suggested that SSR-HRM could distinguish better and was much more accurate than PAGE. Based on different calculating method, sensibility and identifying principles, the result of SSR-HRM derived from one pair of SSR markers might different from sequencing, such as Nos. 11 and 12 accessions. However, the final result of the genetic relationship would tend to be consistent with the increasing number of markers used.

In total, 135 primer pairs were used to analyze the genetic relationship among 32 Chinese bayberry (*Myrica rubra*) accessions through PAGE method [7]. However, 32 *Luffa* specific cultivars could easily be identified by only 19 SSR markers in this study. It indicated that SSR-HRM is a method with relative high resolution, high throughput and efficiency. Compared with sequencing, SSR-HRM requires less money and could obtain nearly the same result in a relatively short period. Therefore, SSR-HRM has become increasingly popular in many crop analyses such as cultivar identification and genotyping [11,15,16].

In this study, 28 accessions belonging to *L. cylindrica* and four accessions belonging to *L. acutangula* were clearly divided into two clusters (Figure 9). Cluster A was *L. cylindrica* with different phenotypes. Both “Lvbaoshi” and “Chunjianlv” were from Lanzhou, Gansu Province. They had high genetic distance coefficient (~0.83), which was mainly relevant with their highly similar phenotype, such as 200–400 g weight, green fruit color, 4 cm diameter and 35–40 cm length per sponge fruit. In addition, “Jipinduanbang” was green color, short length (~18 cm) and thick diameter (~6.5 cm), while “Baimeichunxiang” was almost white color, ~24 cm length and about 6 cm diameter. Thus, they were clearly separated and genetic distance coefficient was only ~0.59. All four cultivars in Cluster B belonged to *L. acutangula*. “Shuangjiannaihan” was from Guangdong Province; however, it had ~0.71 genetic distance coefficient with the local cultivar “Zheda 84”. It suggested that the similar genetic characteristics existed in those two cultivars. The coefficient ranged from 0.11 to 0.86 throughout the result of dendrogram, and the accessions with similar traits were clustered together. Therefore, based on the advantages of HRM and genomic SSR markers, SSR-HRM technology was considered as a rapid, cost effective and high-throughput method for genotyping analyses.

## 4. Materials and Methods

### 4.1. Plant Materials and DNA Extraction

*Luffa* cultivar “Zheda 23” (*Luffa cylindrica* L.) was grown in the Zijingang Campus, Zhejiang University, Hangzhou City, China (30°18’18”N, 120°4’44.4”E) for the genome survey, which possessed the characteristic of typical cultivation. The genomic DNA was extracted from the tender leaves using the DNA Kit (Tiangenbiotech, Beijing, China). The quality and amount of DNA were checked by means of spectrophotometer analysis using Nanodrop 2000. In addition, the DNA of 32 cultivars (Table 7) was also extracted from the tender leaves by DNA kit (Foregene, Chengdu, China).

### 4.2. Genome Sequencing and Sequence Assembly

Following the standard protocol (Illumina, Beijing, China), DNA library with insert size of 220 base pairs (bp) was constructed from randomly fragmented genomic DNA. Sequencing date was produced using the Illumina HiSeq 2500 sequencing platform (Beijing Biomarker Technologies Co., Ltd. Beijing, China). After filtering and correction of the raw data, clean reads were obtained. The high quality reads were then assembled to contigs and scaffolds using SOAPdenovo software (http://soap.genomics.org.cn/soapdenovo.html). All of the clean reads were used to conduct de novo assembly.

### 4.3. Genome Size Estimation, GC Content and Genome Survey

All of the clean data were used for K-mer analysis. K-mer analysis was used to estimate the genome size (Genome size = K-mer count/Peak of the depth distribution) and characters, such as repetitive sequences and heterozygosis. The GC average sequencing depth and content was calculated by the 10-kb non-overlapping sliding windows along the assembled sequence. The characteristics of SSR motif were briefly shown according to the genome survey.

### 4.4. Genomic SSR Marker Development

The Perl script microsatellite searching tool (MISA) (http://pgrc.ipk-gatersleben.de/misa/misa.html) was used to identify microsatellite repeats in *L. cylindrica* genome sequence database. In this study, the SSR loci containing perfect SSR motif repeats of 2–6 nucleotides were only considered. The minimum SSR length criteria were defined as six reiterations for di-nucleotide, and five reiterations for other SSR motif repeats. Eighty primers were designed by Primer Premier 5.0 (Premier Biosoft International, Palo Alto, CA, USA), and all followed the parameters: 18–22 bp primer size, 100–300 bp product length and 50–60 °C annealing temperature. Primers were synthesized by TsingKe Co., Ltd., Hangzhou, China.

### 4.5. Genetic Relationship Analysis by SSR-HRM

We primarily tested two *Luffa* cultivars (“Zheda 23” and “Zheda 83”) for 80 SSR loci. SSR PCR amplification was conducted in a 20 μL volume containing 10 ng of genomic DNA, 2 U Taq DNA polymerase (TaKaRa, Dalian, China), 2.0 mM MgCl_2_, 0.20 mM dNTPs and 0.2 μM each primer. The PCR protocol consisted of a pre-incubation at 95 °C for 300 s, followed by 45 cycles of 95 °C for 10 s, annealing for 10 s at 50 °C, 72 °C for 10 s, and a final extension step of 72 °C for 10 min. PCR reactions were carried out in a thermal cycler C1000 (Bio-Rad, Hercules, California, USA). PCR products were separated on 3% agarose gels at 110 V, then stained with GelRedTM (Biotium, Hayward, CA, USA) and photographed under UV light using Image LabTM software Version 2.0.1 (Bio-Rad, Hercules, CA, USA).

To evaluate the reliability and efficiency of SSR-HRM method, PAGE and sequencing were carried out. As for PAGE, PCR products obtained with the SSR marker ZJULM 50 were run on 6% denaturing polyacrylamide gel at 60 mA constant current and polymorphism was detected by silver staining [34]. GelAnalyzer (Version 2010a) was used to analyze the gel image generated by PAGE. Sequencing of PCR products obtained with the SSR marker ZJULM 50 were sequenced by ABI 3730xl sequencing platform (TsingKe Co., Ltd., Hangzhou, China). The evolutionary relationships analysis (Figure 8) was conducted by Molecular Evolutionary Genetics Analysis version 7.0 (MEGA7). The evolutionary history was inferred using the Neighbor-Joining method [18]. The bootstrap consensus tree inferred from 10,000 replicates was taken to represent the evolutionary history of the taxa analyzed [35]. Branches corresponding to partitions reproduced in less than 50% bootstrap replicates are collapsed. The evolutionary distances were computed using the Maximum Composite Likelihood method [36] and are in the units of the number of base substitutions per site. Codon positions included were 1st + 2nd + 3rd + Noncoding. All positions containing gaps and missing data were eliminated. Evolutionary analyses were conducted in MEGA7 [37].

Nineteen distinct genomic SSR markers were chosen to identify 32 cultivars by Real-Time PCR system using SSR High Resolution Melting method (SSR-HRM). The high resolution melting was carried out by the LightCycler 96 real Real-Time polymerase chain reaction (PCR) System (Roche Diagnostics GmbH, Roche Applied Science, Mannheim, Germany) in a total volume of 20 μL, which containing 10 ng of genomic DNA, 2.0 mM MgCl_2_, 10 μL LightCycler^®^ 480 High Resolution Melting Master Mix with LightCycler^®^ 480 ResoLight Dye (Roche, Basel, Switzerland), 0.2 μM reverse primer and 0.2 μM forward primer. The reactions were subjected to a touchdown PCR thermal protocol consisting of an initial incubation 95 °C for 600 s, then followed by 55 cycles at 95 °C (20 s), 62–50 °C (20 s), and 72 °C (20 s). The annealing temperature decreased by 1 °C per cycle from 62 to 50 °C, and then kept 50 °C in subsequent cycle. The amplification procedure was immediately followed by the high resolution melting steps: 95 °C for 60 s, cooling to 40 °C for 60 s, and then the temperature was rapidly raised to 65 °C. Subsequently, the temperature was raised from 65 to 97 °C with each steps of 0.02 °C for 1 s. After reactions, SSR-HRM raw data were obtained.

LightCycler^®^ 96 (Version 1.1.0.1320 Roche Applied Science, Mannheim, Germany) was used to analyze the SSR-HRM raw data. In the analysis option, both delta*T*m discrimination and curve shape sensitivity were set up to 50% and afterwards the curves were analyzed, then melting curves and difference plots were generated, and finally these curves would be clustered to several genotype groups. The genotype result needed be list strictly with their accessions and markers (Table 5). Each HRM reaction was three repeats and some reactions might fail to perform a HRM process, then some of wrong data would be generated. These wrong data strongly affected the judgment of normal data. Therefore, these wrong data must be removed, and the corresponding genotype number should be encode “0”. Then, the genotype results could be analyzed by traditional software. For example, Nei’s (1973) gene diversity, Polymorphism information content (PIC) was calculated using Power Marker (version 3.25) [38] (http://statgen.ncsu.edu/powermarker/downloads.htm). The genetic similarity coefficient and UPGMA cluster analysis of these accessions (Figure 9) were calculated by NTSYS software (Version 2.10e, New York, NY, USA) [39].

## 5. Conclusions

*Luffa cylindrica* (L.) Roem. had a genome of about 789.97 Mb with little heterozygosity. The genomic SSR combined high resolution melting could be effectively used for genotype relationship analysis of *Luffa* species.

## Figures and Tables

**Table 1:** Statistics of *Luffa cylindrica* L. sequencing data.

Library	Data (Gb)	Depth (×)	Q20 (%)	Q30 (%)
220 bp	43.40	54.94	96.77	91.56

**Table 2:** Statistics of the *Luffa cylindrica* L. genome assembly.

**Scaffold Number**	**Scaffold Length (bp)**	**Scaffold N_50_ (bp)**	**Scaffold N_90_ (bp)**	**Gap total Length (bp)**
1,410,117	885,010,283	807	266	9,841,708
**Contig Number**	**Contig Length (bp)**	**Contig N_50_ (bp)**	**Contig N_90_ (bp)**	**GC Content (%)**
1,913,731	875,168,575	525	236	34.34

The N_50_ of scaffolds and contigs was calculated by ordering all sequences, then adding the lengths from the longest to shortest until the added length exceeded 50% of the total length of all sequences. N_90_ is similarly defined.

**Table 3:** Simple sequence repeat types detected in the *Luffa cylindrica* L. sequences.

Searching Item	Number	Percentage
Total number of sequences examined	2,697,125	–
Total size of examined sequences (bp)	1,064,011,890	–
Total number of identified SSRs	431,234	100.00%
Number of SSRs containing sequences	218,940	50.77%
Number of sequences containing more than 1 SSR	74,313	17.23%
Number of SSRs present in compound formation	47,595	11.04%
Mono-nucleotide	306,140	70.99%
Di-nucleotide	78,655	18.24%
Tri-nucleotide	38,823	9.00%
Tetra-nucleotide	5737	1.33%
Penta-nucleotide	1196	0.28%
Hexa-nucleotide	683	0.16%

**Table 4:** Characteristics of 19 SSR markers in present study.

Locus	GenBank Accession	Repeat Motif	Primer Sequence (5′–3′)	Gene Diversity	PIC
ZJULM 3	KY983987	(TTTTAT)6	Forward: CGTGTCTTCGGGATAATA Reverse: CGAAGCATCTTTTACCAT	0.6032	0.5556
ZJULM 36	KY983988	(CTTCCA)6	Forward: CTGAGCAGTCTAACACCCAT Reverse: TGTGCGAACAAGGAAGGA	0.4141	0.3874
ZJULM 39	KY983989	(AAAAAT)5	Forward: GCGAGTATAGCTCAACGG Reverse: TTCCAAAATCCAAACCAA	0.3673	0.3484
ZJULM 41	KY983990	(TGATGG)5	Forward: TCGTTGGTGTTGTAGGGTTT Reverse: GAGGACGAATTGGAAGGAGT	0.6900	0.6404
ZJULM 45	KY983991	(TTTTA)5	Forward: AATTCCCAGGTAATGTTATG Reverse: GTCGGCTTGTTTCTTCTC	0.6563	0.5888
ZJULM 46	KY983992	(AAGAG)5	Forward: CCCGCAGTGTTAAGTTTC Reverse: CCTGCCATGTTTGTTCTC	0.4019	0.3756
ZJULM 48	KY983993	(AAAAT)5	Forward: TAGAAAGGAAAGGAGGAA Reverse: TTCAAGAGTTCAGGGTTT	0.6240	0.5527
ZJULM 50	KY983994	(TAAGA)5	Forward: TTCTCCAAATAAGCCACT Reverse: AGAATCTCCTACCCGTTT	0.8137	0.7896
ZJULM 51	KY983995	(TGGTTG)6	Forward: CCAGTCCAGGAGAAAGGG Reverse: GAGGCACAACCACAACCA	0.6505	0.6129
ZJULM 53	KY983996	(GAAAGT)5	Forward: GCGAAGAGGAGCGAAGAA Reverse: ATTGGCAATGCAATGAGG	0.3889	0.3613
ZJULM 55	KY983997	(CCAGCA)5	Forward: GATAATGGAAATAAACCACCCT Reverse: GCCACAGACCCTACTTGAGA	0.7813	0.7506
ZJULM 59	KY983998	(CCACCT)7	Forward: CGTGTAGGCTAGGGTCAC Reverse: CTCCACCACTTCATTTGTAT	0.5898	0.5418
ZJULM 66	KY983999	(TTTTGT)5	Forward: AAGATCGGTTTGGGAGGA Reverse: TGGCAGTTTCAGGCAGTC	0.6468	0.6156
ZJULM 67	KY984000	(GTTTTT)5	Forward: GGGATATTGCGGTGGAGT Reverse: GGTTAGGTGGCGTTCGTC	0.7111	0.6637
ZJULM 71	KY984001	(ATTTTT)5	Forward: AGTTCCCTGAGCAGATAC Reverse: CTAAATCAACAACATCCCT	0.5729	0.5439
ZJULM 73	KY984002	(TGGTTG)6	Forward: TTGAGCCTGAGGGATAGA Reverse: GGATGCTGCTGATAAGTG	0.7041	0.6798
ZJULM 77	KY984003	(AGAGAA)5	Forward: TTCGGTCATTTGATTTCG Reverse: TTCGTGGAAGAACCCTCT	0.1913	0.1730
ZJULM 78	KY984004	(AGTTCC)5	Forward: GAACATCCCAGGAAATGC Reverse: GCCAGACGAGGAAGAACA	0.2246	0.2096
ZJULM 79	KY984005	(GAAAAA)5	Forward: GAGGAGATGGTGAGGGAG Reverse: AACGGATTGCTGATGTGA	0.6806	0.6427
Mean	–	–	–	0.5638	0.5281

Shown for each primer pair are the repeat motif, primer sequences, gene diversity, and polymorphism information content (PIC).

**Table 5:** Result of genotype group number obtained with 19 SSR markers.

Accession Number	ZJU *Luffa* Marker																		
	3	36	39	41	45	46	48	50	51	53	55	59	66	67	71	73	77	78	79
1	1	3	0	2	2	1	2	2	4	3	6	2	3	3	1	0	1	1	1
2	1	1	1	2	2	1	2	3	0	3	1	2	1	1	1	2	1	1	1
3	3	1	1	2	2	1	2	1	1	1	5	2	1	1	1	2	1	1	1
4	4	1	1	2	2	1	2	6	0	1	5	1	1	1	1	2	1	1	1
5	1	1	1	1	2	1	1	0	1	1	6	1	3	1	1	5	1	1	1
6	5	2	2	3	1	4	2	8	3	1	2	3	5	4	2	7	1	2	4
7	0	2	2	3	3	3	2	8	3	1	2	3	4	4	2	0	1	3	4
8	3	2	4	3	0	4	2	8	5	2	2	3	4	5	2	8	1	2	5
9	0	2	2	3	4	4	2	8	5	4	2	3	5	4	2	1	1	2	5
10	0	1	0	1	1	1	2	1	1	1	1	2	3	3	1	6	1	1	3
11	0	1	1	1	1	1	1	1	1	1	4	2	1	3	1	6	1	1	3
12	0	3	1	1	2	1	1	7	1	1	1	2	6	3	1	1	1	1	3
13	1	1	1	1	1	1	1	5	1	1	5	1	1	3	1	1	1	1	2
14	0	1	1	0	2	0	1	3	2	1	4	1	1	2	1	1	1	1	2
15	0	1	1	1	1	2	1	1	1	1	4	4	1	1	1	1	1	1	2
16	0	1	1	1	1	2	1	1	1	1	1	1	1	1	1	1	1	1	3
17	2	1	1	0	2	0	3	1	1	1	4	0	1	2	3	4	0	1	0
18	0	1	1	4	3	2	1	1	2	1	1	1	1	1	0	0	1	1	1
19	2	1	1	5	1	1	1	5	1	1	3	4	1	1	0	1	2	1	1
20	2	1	1	0	1	1	1	6	1	1	3	1	2	2	4	0	1	1	1
21	2	1	3	2	3	1	1	1	1	1	1	0	1	1	6	5	1	1	1
22	2	1	1	0	0	1	0	5	0	0	1	0	2	1	0	2	0	1	0
23	1	1	1	1	0	1	0	1	2	1	3	1	1	2	7	1	1	1	0
24	0	1	1	0	0	1	3	2	4	1	3	1	0	2	5	1	2	1	1
25	1	1	0	0	1	1	0	4	0	2	1	1	1	1	0	0	1	1	0
26	0	1	1	0	0	0	0	2	1	2	1	1	3	0	0	3	2	1	0
27	1	1	0	1	2	1	0	2	2	2	2	1	0	0	0	3	0	1	0
28	1	4	1	0	3	1	0	2	2	1	2	1	2	2	1	1	0	1	2
29	1	1	1	0	0	1	4	4	1	1	3	1	1	2	0	1	1	1	0
30	1	1	1	0	1	1	1	2	1	1	1	1	2	1	0	1	1	1	0
31	1	4	1	0	0	1	0	2	2	0	1	1	1	0	1	0	1	1	1
32	1	1	5	0	0	1	3	4	6	1	2	1	0	2	1	1	1	1	1

Accessions were marked with their genotype group number after SSR-HRM analyses. “0” means invalid data.

**Table 6:** Genomic SSR markers designed.

Marker	GenBank Accession	Primer sequence (5′–3′)(Forward)	Primer sequence (5′–3′)(Reverse)
ZJULM 1	MF677780	AAATTGGGTATCCATCTC	CATAAAACTTCCGTGAAA
ZJULM 2	MF677781	TATTTGGTCCAACAATAG	TTGAAAGTTCAATAAACC
ZJULM 4	MF677782	CTTTGGGCTTCTTCACTA	TTTGGGTGAAAGTTTTGT
ZJULM 5	MF677783	TCAACACTCTGCCAATTG	AGCCCCATGAACATAAAA
ZJULM 6	MF677784	TAAAAGTTCATTCATTACAC	GACATAACAAAATAGGATAA
ZJULM 7	MF677785	ATCTAAAATAAATTAACGGG	CAAATTTGGTTGAATTTACA
ZJULM 8	MF677786	ATTTGATTCGATGCTACC	GAGCTCTTCGGAATTTTA
ZJULM 9	MF677787	AAAGAAGCATAATCCCTT	TAACCTGCAATTCAATGT
ZJULM 10	MF677788	ATGGGAGTTGGGCTATTT	ATCAGCAGCAGTGTTTGG
ZJULM 11	MF677789	TCTTCCTCCCTCTTATCC	TCTGAATGGGGTTGGTTT
ZJULM 12	MF677790	GTGTTTGGTTATGAATTT	CCTTATAATTTCAATTCC
ZJULM 13	MF677791	TCTCCCTCCCTCTTGCTC	TGTAAACTTAACCCAAACCTC
ZJULM 14	MF677792	TTCTTCTCAGGCACTCCA	CACAAAGTACCAAGGTGG
ZJULM 15	MF677793	TGTTTGGATCTAAAGAAA	TAAACAACATGGATGAAT
ZJULM 16	MF677794	CTCTATGAGGTCTGTGGGAGA	GAGCTAAGCCCCAAAATC
ZJULM 17	MF677795	TTGTCTTTACTATTGGGA	ATTATCAAACATCCACAA
ZJULM 18	MF677796	TCAGGATTGTTAAGCCAGTT	CAATGACCAGCAATGACC
ZJULM 19	MF677797	GCACCTAAGCCAACCAAC	GGACAATGCATGTCACGA
ZJULM 22	MF677798	GAGAAGAAGACTCTGGGG	AAGAAAAGTGAAATCCCA
ZJULM 23	MF677799	GAAAAGTCGTTGACAACA	CAATTTCGTTTGAATGTT
ZJULM 24	MF677800	CGAATGTTAAAGAAACTT	CGAATGTTAAAGAAACTT
ZJULM 25	MF677801	AAAAGTCGTTGACAACAT	TACAATTTCGTTTGAATG
ZJULM 26	MF677802	AAACAGTTTCCCTTACCA	AATATCGTGGAGGTTGTC
ZJULM 27	MF677803	GGACACCAAAGTAAACATGC	CTAGTTTCATCAATTCCAAG
ZJULM 28	MF677804	ACTTGCTTATCAGAGTGGCA	ATGTTTGTCGGTAATGTTCG
ZJULM 29	MF677805	CCACCTGTAATGTTATCCAT	ATTTTGGTACGTTATCTGCT
ZJULM 30	MF677806	AGCAACTAAAATGAGGTAAA	TATTGATGGCATCCATCCTG
ZJULM 31	MF677807	GCCAACTCATAACAAGAATC	TAATCACCAACACCTTATTC
ZJULM 32	MF677808	GAAATGTGAAATCCCACG	TGGACGGAGTAGAGGTGA
ZJULM 33	MF677809	TAGCCGTTCGTTTTCATT	CACCGACATTCTAAATCCTG
ZJULM 34	MF677810	CATGGCGGCTATGAAGGC	TCCGCACAGTGACAGAGTGGT
ZJULM 35	MF677811	TTATGTCTGTCCCGTTCA	ATACCTTATCTTTGTGCC
ZJULM 38	MF677812	GGGGAGAAATAAGAAATAG	TTCGCTTCGTGGTGTTGG
ZJULM 40	MF677813	TATCCAATAAGCTTGAAG	AAACTATCGCATGTAATG
ZJULM 43	MF677814	CTACCCGTGAGAATTTGA	CACTACTTCCACCCACAA
ZJULM 48	MF677815	TAGAAAGGAAAGGAGGAA	TTCAAGAGTTCAGGGTTT
ZJULM 49	MF677816	GAGAAAGATAATTGAAAGGGAT	GTGCTGCCATACGGTTAG
ZJULM 52	MF677817	ATCTAAAATTTAAAGGGG	TAGACCATAATACCCCTT
ZJULM 54	MF677818	TGTTGTTATGAATCGGTGAA	TAGGCAAAGGAAAGTTGG
ZJULM 56	MF677819	TGGCGGCGGAGCAGTGAA	ACCACCCGTAGGGCGTGTCC
ZJULM 57	MF677820	TTCTTCTCCCTCTTTGCT	ACAGTCACCGCCTCATAT
ZJULM 58	MF677821	GTATCGTATCGGGTGCCT	TTCCTTTCCACATGCCTC
ZJULM 60	MF677822	ATTTCTGTTAATTTGGTTCC	CAATCGAATAAAAGGTCAA
ZJULM 62	MF677823	TTTTCAAAGTTCAAGGAC	TTAGTGTCACGTCAGCAT
ZJULM 63	MF677824	CAGGCGAAGCAAAGGATT	TGATGGTCTGACGGAGGC
ZJULM 64	MF677825	TTTGTCACAATCCCACCT	GAATACGCAGCCTTCTTT
ZJULM 65	MF677826	AGAATGATTTACCCGTAG	AGAGGAGGAACTTTTGAT
ZJULM 68	MF677827	CCCCTCCCCTCCAAAATA	TTGCCCAGGAACGAACTT
ZJULM 69	MF677828	TCATTCCTACCGAAAGTA	AACGGACCCTTATACTTG
ZJULM 70	MF677829	AAGCGGGAGCTAAGAATG	GCTGGAATGTTGGGAGAA
ZJULM 72	MF677830	ACACCGTAACAGATCAAA	CTCATTCTTTCCCTTTCT
ZJULM 74	MF677831	ATCTAAAATTTAAAGGGG	TAGACCATAATACCCCTT
ZJULM 75	MF677832	TGTTGTTATGAATCGGTGAA	TAGGCAAAGGAAAGTTGG
ZJULM 76	MF677833	AACCCACAGAATAAAGATG	GAAGAAGCTCCTACCTGA
ZJULM 80	MF677834	TCAATGCCAGTGTCTCAA	GCTTCTTATTGGACCTATTT

**Table 7:** The 32 *Luffa* accessions included in present study.

No.	Accession	Region	No.	Accession	Region
1	Zheda 1	Hangzhou, Zhejiang	17	Zheda 7	Hangzhou, Zhejiang
2	Zheda 2	Hangzhou, Zhejiang	18	Zheda 8	Hangzhou, Zhejiang
3	Zheda 23	Hangzhou, Zhejiang	19	Zheda h101	Hangzhou, Zhejiang
4	Zheda 56	Hangzhou, Zhejiang	20	Zheda h100	Hangzhou, Zhejiang
5	Zheda 10	Hangzhou, Zhejiang	21	Zheda 57N	Hangzhou, Zhejiang
6	Zheda 81	Hangzhou, Zhejiang	22	Zheda 72	Hangzhou, Zhejiang
7	Zheda 83	Hangzhou, Zhejiang	23	Zheda 22	Hangzhou, Zhejiang
8	Zheda 84	Hangzhou, Zhejiang	24	Zheda 70	Hangzhou, Zhejiang
9	Shuangjiannaihan1	Heshan, Guangdong	25	Zheda 5	Hangzhou, Zhejiang
10	Jizaoxiangyu	Changsha, Hunan	26	Zheda h6	Hangzhou, Zhejiang
11	Jinxiuzaojia	Lanzhou, Gansu	27	Zheda h7	Hangzhou, Zhejiang
12	Sanbier	Changde, Hunan	28	Zheda 85	Hangzhou, Zhejiang
13	Baimeichunxiang	Changsha, Hunan	29	Zheda 86	Hangzhou, Zhejiang
14	Jipinduanbang	Changsha, Hunan	30	Zheda 87	Hangzhou, Zhejiang
15	Lvbaoshi	Lanzhou, Gansu	31	Zheda 88	Hangzhou, Zhejiang
16	Chunjianlv	Lanzhou, Gansu	32	Zheda h4	Hangzhou, Zhejiang

## Supplementary Materials

All SSR Markers developed based on de novo genome assembly sequence of *L. cylindrica* have been deposited in LabArchives at http://dx.doi.org/10.6070/H4QF8QZQ. All SSR-HRM result, including normalized melting curves, different plots and genotype group result have also been deposited in LabArchives at http://dx.doi.org/10.6070/H4KS6PNQ. All other data supporting the conclusions of this article are included within the article and its additional files.

## Abbreviations

SSR-HRM	Microsatellite high resolution melting
NGS	Next-generation sequencing
SSR	Microsatellite
UPGMA	Unweighted pair group method analysis
EST	Expressed sequence tag
ZJULM	Zhejiang University *luffa* marker
MISA	Microsatellite searching tool
GC	Guanine pluscytosine
NCBI	National center for biotechnology information
HRM	High resolution melting
PCR	Polymerase chain reaction
MEGA	Molecular evolutionary genetics analysis
PIC	Polymorphism information content
PAGE	Polyacrylamide gel electrophoresis

## References

[1] Wu H.B., He X.L., Gong H., Luo S.B., Li M.Z., Chen J.Q., Zhang C.Y., Yu T., Huang W.P., Luo J.N. (2016). Genetic linkage map construction and QTL analysis of two interspecific reproductive isolation traits in sponge gourd. Front. Plant. Sci..

[2] Joshi B.K., Kc H.B., Tiwari R.K., Ghale M., Sthapit B.R. (2004). Descriptors for sponge gourd (*Luffa cylindrica* (L.) Roem.). https://idl-bnc-idrc.dspacedirect.org/bitstream/handle/10625/31459/122785.pdf?sequence=1.

[3] Partap S., Kumar A., Sharma N.K., Jha K.K. (2012). *Luffa cylindrica*: An important medicinal plant. J. Nat. Prod. Plant Resour..

[4] Sheng Z., Jin H., Zhang C.F., Guan Y.J., Ying Z. (2007). Genetic analysis of fruit shape traits at different maturation stages in sponge gourd. J. Zhejiang Univ. Sci. B.

[5] Lu M., An H.M., Li L.L. (2016). Genome survey sequencing for the characterization of the genetic background of *Rosa roxburghii* tratt and leaf ascorbate metabolism genes. PLoS ONE.

[6] Zhou W., Hu Y.Y., Sui Z.H., Fu F., Wang J.G., Chang L.P., Guo W.H., Li B.B. (2013). Genome survey sequencing and genetic background characterization of *Gracilariopsis lemaneiformis* (Rhodophyta) based on next-generation sequencing. PLoS ONE.

[7] Jiao Y., Jia H.M., Li X.W., Chai M.L., Jia H.J., Chen Z., Wang G.Y., Chai C.Y., van de Weg E., Gao Z.S. (2012). Development of simple sequence repeat (SSR) markers from a genome survey of Chinese bayberry (*Myrica rubra*). BMC Genomics..

[8] Wu H.B., Gong H., Liu P., He X.L., Luo S.B., Zheng X.M., Zhang C.Y., He X.M., Luo J.N. (2014). Large-scale development of EST-SSR markers in sponge gourd via transcriptome sequencing. Mol. Breed..

[9] Wang Y.W., Samuels T.D., Wu Y.Q. (2011). Development of 1,030 genomic SSR markers in switchgrass. Theor. Appl. Genet..

[10] Wilhelm J., Pingoud A., Hahn M. (2003). Validation of an algorithm for automatic quantification of nucleic acid copy numbers by real-time polymerase chain reaction. Anal. Biochem..

[11] Ganopoulos I., Argiriou A., Tsaftaris A. (2011). Microsatellite high resolution melting (SSR-HRM) analysis for authenticity testing of protected designation of origin (PDO) sweet cherry products. Food Control.

[12] Wittwer C.T. (2009). High-resolution DNA melting analysis: Advancements and limitations. Hum. Mutat..

[13] Wittwer C.T., Reed G.H., Gundry C.N., Vandersteen J.G., Pryor R.J. (2003). High-resolution genotyping by amplicon melting analysis using LCGreen. Clin. Chem..

[14] Tindall E.A., Petersen D.C., Woodbridge P., Schipany K., Hayes V.M. (2009). Assessing high-resolution melt curve analysis for accurate detection of gene variants in complex DNA fragments. Hum. Mutat..

[15] Bosmali I., Ganopoulos I., Madesis P., Tsaftaris A. (2012). Microsatellite and DNA-barcode regions typing combined with high resolution melting (HRM) analysis for food forensic uses: A case study on lentils (*lens culinaris*). Food Res. Int..

[16] Xanthopoulou A., Ganopoulos I., Koubouris G., Tsaftaris A., Sergendani C., Kalivas A., Madesis P. (2014). Microsatellite high-resolution melting (SSR-HRM) analysis for genotyping and molecular characterization of an *Olea europaea* germplasm collection. Plant Genet. Resour..

[17] Chitsaz H., Yee-Greenbaum J.L., Tesler G., Lombardo M.J., Dupont C.L., Badger J.H., Novotny M., Rusch D.B., Fraser L.J., Gormley N.A. (2011). Efficient *de novo* assembly of single-cell bacterial genomes from short-read data sets. Nat. Biotechnol..

[18] Saitou N., Nei M. (1987). The neighbor-joining method—A new method for reconstructing phylogenetic trees. Mol. Biol. Evol..

[19] Nei M. (1972). Genetic distance between populations. Am. Nat..

[20] Garcia-Mas J., Benjak A., Sanseverino W., Bourgeois M., Mir G., Gonzalez V.M., Henaff E., Camara F., Cozzuto L., Lowy E. (2012). The genome of melon (*Cucumis melo* L.). Proc. Natl. Acad. Sci. USA.

[21] Huang S.W., Li R.Q., Zhang Z.H., Li L., Gu X.F., Fan W., Lucas W.J., Wang X.W., Xie B.Y., Ni P.X. (2009). The genome of the cucumber, *Cucumis sativus* L.. Nat. Genet..

[22] Hou S.Y., Sun Z.X., Bin L.H., Xu D.M., Wu B., Zhang B., Wang X.C., Han Y.H., Zhang L.J., Qiao Z.J. (2016). Genetic diversity of buckwheat cultivars (*Fagopyrum tartaricum* gaertn.) assessed with SSR markers developed from genome survey sequences. Plant Mol. Biol. Rep..

[23] Cheung M.S., Down T.A., Latorre I., Ahringer J. (2011). Systematic bias in high-throughput sequencing data and its correction by beads. Nucleic Acids Res..

[24] Aird D., Ross M.G., Chen W.S., Danielsson M., Fennell T., Russ C., Jaffe D.B., Nusbaum C., Gnirke A. (2011). Analyzing and minimizing PCR amplification bias in illumina sequencing libraries. Genome Biol..

[25] Bentley D.R., Balasubramanian S., Swerdlow H.P., Smith G.P., Milton J., Brown C.G., Hall K.P., Evers D.J., Barnes C.L., Bignell H.R. (2008). Accurate whole human genome sequencing using reversible terminator chemistry. Nature.

[26] Hirakawa H., Okada Y., Tabuchi H., Shirasawa K., Watanabe A., Tsuruoka H., Minami C., Nakayama S., Sasamoto S., Kohara M. (2015). Survey of genome sequences in a wild sweet potato, *Ipomoea trifida* (H.B.K.) G. Don. DNA Res..

[27] Xu X., Pan S.K., Cheng S.F., Zhang B., Mu D.S., Ni P.X., Zhang G.Y., Yang S., Li R.Q., Wang J. (2011). Genome sequence and analysis of the tuber crop potato. Nature.

[28] Werren J.H., Richards S., Desjardins C.A., Niehuis O., Gadau J., Colbourne J.K., Beukeboom L.W., Desplan C., Elsik C.G., Grimmelikhuijzen C.J.P. (2010). Functional and evolutionary insights from the genomes of three parasitoid *Nasonia* species. Science.

[29] Zhao H.S., Yang L., Peng Z.H., Sun H.Y., Yue X.H., Lou Y.F., Dong L.L., Wang L.L., Gao Z.M. (2015). Developing genome-wide microsatellite markers of bamboo and their applications on molecular marker assisted taxonomy for accessions in the genus *Phyllostachys*. Sci. Rep..

[30] Shi J.Q., Huang S.M., Zhan J.P., Yu J.Y., Wang X.F., Hua W., Liu S.Y., Liu G.H., Wang H.H. (2014). Genome-wide microsatellite characterization and marker development in the sequenced *Brassica* crop species. DNA Res..

[31] Temnykh S., DeClerck G., Lukashova A., Lipovich L., Cartinhour S., McCouch S. (2001). Computational and experimental analysis of microsatellites in rice (*Oryza sativa* L.): Frequency, length variation, transposon associations, and genetic marker potential. Genome Res..

[32] Zhou X.J., Dong Y., Zhao J.J., Huang L., Ren X.P., Chen Y.N., Huang S.M., Liao B.S., Lei Y., Yan L.Y. (2016). Genomic survey sequencing for development and validation of single-locus SSR markers in peanut (*Arachis hypogaea* L.). BMC Genomics.

[33] Katti M.V., Ranjekar P.K., Gupta V.S. (2001). Differential distribution of simple sequence repeats in eukaryotic genome sequences. Mol. Biol. Evol..

[34] Bassam B.J., Caetanoanolles G., Gresshoff P.M. (1991). Fast and sensitive silver staining of DNA in polyacrylamide gels. Anal. Biochem..

[35] Felsenstein J. (1985). Confidence-limits on phylogenies—An approach using the bootstrap. Evolution.

[36] Tamura K., Nei M., Kumar S. (2004). Prospects for inferring very large phylogenies by using the neighbor-joining method. Proc. Natl. Acad. Sci. USA.

[37] Kumar S., Stecher G., Tamura K. (2016). Mega7: Molecular evolutionary genetics analysis version 7.0 for bigger datasets. Mol. Biol. Evol..

[38] Liu K., Muse S.V. (2005). Powermarker: An integrated analysis environment for genetic marker analysis. Bioinformatics.

[39] Powell W., Morgante M., Andre C., Hanafey M., Vogel J., Tingey S., Rafalski A. (1996). The comparison of RFLP, RAPD, AFLP and SSR (microsatellite) markers for germplasm analysis. Mol. Breed..

